# Change in Tinnitus Severity After an Online Self-Paced Tinnitus Course: A Retrospective Cohort Study in Acute and Chronic Tinnitus Patients

**DOI:** 10.3390/jcm14041166

**Published:** 2025-02-11

**Authors:** Annemarie van der Wal, Frank Lobbezoo, Roel van Gorkum, Naichuan Su, Hans Korfage

**Affiliations:** 1 Department of Orofacial Pain and Disfunction, Academic Centre for Dentistry Amsterdam (ACTA), University of Amsterdam and Vrije Universiteit (VU) University Amsterdam, 1081 HV Amsterdam, The Netherlands; f.lobbezoo@acta.nl (F.L.); j.korfage@acta.nl (H.K.); 2 Department of Rehabilitation Sciences and Physiotherapy, Faculty of Medicine and Health Sciences, University of Antwerp, 2000 Antwerp, Belgium; 3 Department of Orofacial Pain and Jaw Function, Faculty of Odontology, Malmö University, 21119 Malmö, Sweden; 4 Still Tinnitus, 1015 AE Amsterdam, The Netherlands; roel@stilltinnitus.com; 5 Department of Oral Public Health, Academic Centre for Dentistry Amsterdam (ACTA), University of Amsterdam and Vrije Universiteit (VU) University Amsterdam, 1081 HV Amsterdam, The Netherlands; n.su@acta.nl

**Keywords:** tinnitus, eHealth, cognitive behavioral therapy, tinnitus retraining therapy, acceptance and commitment therapy, mindfulness

## Abstract

**Background:** Tinnitus can significantly impact a patient’s quality of life. As no evidence-based curative treatments exist, therapies such as cognitive behavioral therapy, tinnitus retraining therapy, acceptance and commitment therapy, and mindfulness-based interventions aim to minimize tinnitus severity and have been shown effective. Since traditional delivery can be costly and time-consuming and often has limited accessibility, therapies might also be provided via eHealth. This study investigates the change in tinnitus severity measured by the Tinnitus Functional Index (TFI) score after participation in an online self-paced tinnitus (“Still Tinnitus”) course. The secondary aim was to identify predictors for the clinically relevant improvement after participation in this course. **Methods:** This retrospective record study included patients from Still Tinnitus course between March 2023 and July 2024. Patients were recruited via the Still Tinnitus website. Differences in the TFI scores from baseline and after completing the fifth (last) module of the course were calculated to investigate the change in tinnitus over time. Multivariate logistic analyses were performed to identify the possible predictors for the clinically relevant improvement after completion of the Still Tinnitus course. **Results:** In total, 122 patients were included in the study. The analysis revealed a clinically relevant reduction in the TFI score of 27.2 points. Multiple regression analyses showed that the “duration of the tinnitus” (OR 5.0; 95%CI: 1.537–16.240; *p* = 0.007) and “female sex” (OR 1.9; 95%CI 0.111–7.637; *p* = 0.030) are predictors for a clinically relevant improvement. **Conclusions:** In a convenience sample of tinnitus patients, the Still Tinnitus course may contribute to a clinically relevant reduction in tinnitus severity. A shorter duration of tinnitus and female sex were identified as significant predictors.

## 1. Introduction

Tinnitus is the conscious awareness of a sound for which there is no identifiable corresponding external acoustic source [1]. Globally, around 740 million adults have tinnitus, and more than 120 million people perceive it as a major problem [2]. Tinnitus can be very debilitating, and it can greatly harm a patient’s daily quality of life [3,4]. It can affect their psychological well-being, work, social life, and physical functioning [5,6]. Recently, an international group of experts in the field of tinnitus proposed the term ‘tinnitus disorder’, which refers to ‘tinnitus plus tinnitus-associated emotional distress and functional disability’ [1].

Various underlying diseases and conditions have been described that might influence or trigger tinnitus. The main risk factor is hearing loss, but other risk and influencing factors such as noise trauma, temporomandibular disorders, psychological factors (like stress, anxiety, and depression disorder), and head injury have been reported as well [7,8,9,10]. Furthermore, otologic diseases, like acoustic neuroma and Meniere’s disease, and various ototoxic medications can trigger tinnitus [11]. However, in most cases, causation cannot be identified. Therefore, tinnitus is considered a complex phenomenon with many different influencing factors and mechanisms that play a role in its generation and continuation.

To date, no evidence-based curative treatments exist [12]. Current treatments aim to minimize distress and thereby improve quality of life. Significant positive outcomes have been reported for cognitive behavioral therapy (CBT), tinnitus retraining therapy (TRT), acceptance and commitment therapy (ACT), and mindfulness-based interventions (MBI) [13,14,15,16,17]. CBT is recommended by various guidelines [18]. All of these psychotherapeutic approaches allow negative thoughts, emotions, and behaviors to be identified and altered through education and counselling [19]. Greater knowledge and acquired coping strategies can help reduce fear and mental distress. In this way, patients will learn to stop paying attention to stimuli that are irrelevant or meaningless and the tinnitus will no longer affect their quality of life. This concept is called tinnitus habituation [20,21]. 

Early treatment is suggested to be beneficial in managing tinnitus and minimizing the impact on quality of life [22,23,24]. However, traditional delivery (through direct physical contact with a therapist) can be costly and time-consuming [25]. Additional treatment barriers, such as long waiting lists, scarcity of trained professionals, and travel distances, further complicate habituation and lead to high dropout rates [26]. As a result, a large portion of patients are still left untreated [27]. Hence, an urgent need exists to improve the accessibility of interventions [28]. 

For this reason, the interest in the potential of eHealth and self-controlled individual therapies has increased [29]. Promising results have been reported for eHealth-mediated CBT by Beukes et al. [30], Hesser et al. [31], and Bardy et al. [32]. Also, the use of online TRT, ACT, and MBI might be effective for the reduction in tinnitus disorder [16,33,34]. However, according to a review of Demoen et al. [26], there is still low-to-moderate quality evidence that eHealth therapy is effective to reduce tinnitus severity and distress.

The newly developed online therapy approach “Still Tinnitus” combines the abovementioned four online management strategies into a single self-paced course. Given prior evidence supporting the effectiveness of internet-based tinnitus interventions, we hypothesize that combining the abovementioned psychological therapies in an online self-paced tinnitus format will result in a clinically relevant reduction in the impact of tinnitus on a patient’s quality of life.

The present study aims to investigate the change in tinnitus severity measured by the Tinnitus Functional Index (TFI) score after participation in the Still Tinnitus course. Additionally, this study seeks to identify predictors of positive outcome for tinnitus patients. Our hypothesis is that younger patients with a shorter duration of tinnitus are more likely to experience greater benefits from the treatment. We reason that younger patients are more accustomed to utilizing and learning from eHealth compared to older adults [35]. Moreover, previous studies have shown that a shorter disease duration predicts a positive treatment outcome [36,37].

## 2. Methods

### 2.1. Design

A retrospective cohort study was conducted using the database from the Still Tinnitus organization (Amsterdam, The Netherlands). This study is approved by the ACTA Institutional Review Board under protocol number 2024-56913. Still Tinnitus provided anonymization of the patients’ data. Only the necessary data were communicated to the researchers via a secure file. Patients were given a unique number. No names or personal details were shared. As a result, the data were not traceable to an individual.

### 2.2. Patients

We selected a convenience sample out of tinnitus patients who participated in the Still Tinnitus course between March 2023 and July 2024. Anyone could purchase and start the course immediately via the website “www.stilltinnitus.com”, accessible via Google and advertised on various social media platforms (i.e., LinkedIn, YouTube).

The eligibility criteria used to select the patients were as follows: (1) being at least 18 years of age, (2) having completed all five modules of the Still Tinnitus course, (3) having a TFI score of 32 and up, i.e., a “moderate problem” to a “very big problem”, and (4) having completed the TFI questionnaire at baseline and after completing the course. At the beginning of the course, patients were invited to consent to the use of their data for scientific purposes. Upon obtaining consent, the data were securely stored in the Still Tinnitus database. Patients were asked to provide demographic and clinical information including age, sex, and duration of their tinnitus symptoms. For tinnitus duration, patients selected from predefined options: “one week”, “one month”, “between 1 and 3 months”, “between 3 and 6 months”, “between 6 and 12 months”, and “more than 12 months”. The number of days between initiation and completion of the course was determined by calculating the number of days between the TFI baseline test and their last TFI test.

### 2.3. Intervention

The selected intervention was the Still Tinnitus course. Patients independently completed the e-learning course consisting of 60 video lectures, 40 workbook assignments, a mobile app, and quizzes. The course is based on 4 methods: CBT, TRT, ACT, and MBI. The content emphasizes in-depth tinnitus education and provides coping and management tools. In this way, Still Tinnitus aims to improve the patient’s quality of life. Components include identifying and challenging negative thoughts and feelings, behavior activation, cognitive restructuring, habituation, relaxation, meditation, sound therapy, and mindfulness. The course is structured into 5 modules.

In module 1, patients are encouraged to visit their GP/ENT specialist and get their hearing tested. They receive essential ‘first aid’ tips and start TRT sound therapy through the app. Detailed information is provided on the causes and mechanisms of tinnitus. This increases their understanding of their condition and aims to provide initial relief.

In module 2, the scientific basis for the course and concepts like habituation and neuroplasticity are outlined. This section is designed to give patients hope by demonstrating that it is possible to manage and improve their tinnitus severity.

Module 3 focuses on calming the nervous system through techniques from Mindfulness-Based Stress Reduction (MBSR) and tinnitus retraining therapy (TRT). These methods help patients address underlying factors to reduce stress and anxiety, improve sleep, and find peace with their tinnitus sound by using specially designed meditations available through the app.

In module 4, cognitive behavioral therapy (CBT) and acceptance and commitment therapy (ACT) tools are introduced to help patients shift their thoughts and feelings about the sound. Using special worksheets, the patients gradually reintroduce themselves to activities they had previously avoided due to tinnitus and start to reclaim their life.

In module 5, patients create a personalized plan summarizing their insights and strategies, empowering them to continue their habituation journey independently. Additionally, an optional coaching call with either the developer of the course (RvG) or a psychologist could be booked. A video tour of the digital course environment is available here (https://stilltinnitus.com/course-tour, accessed on 12 January 2025).

### 2.4. Outcome Measurement

The validated Tinnitus Functional Index (TFI) was selected as the outcome variable for this study. The TFI is a clinical self-reporting questionnaire for chronic, intrusive tinnitus and can be used to assess changes in tinnitus severity [38]. The psychometric properties of the Dutch version of the TFI questionnaire are comparable to the original TFI developed by Meikle et al. [38,39]. The Dutch TFI response is requested at baseline and after finishing the fifth (last) module. The TFI is specifically designed to measure treatment effects in addition to the severity and impact of tinnitus [40,41]. The questionnaire consists of 25 questions covering eight tinnitus domains: (1) intrusive, (2) sense of control, (3) mental, (4) sleep, (5) hearing, (6) relaxation, (7) quality of life, and (8) emotions. Each question is scored on a Likert scale from zero to ten, in which the higher scores indicate higher levels of negative impact of tinnitus. Eight separate subscale scores and a total score are calculated.

Total scores between 0 and 17 are interpreted as “not a problem”, total scores between 18 and 31 as “small problem”, total scores between 32 and 53 as a “moderate problem”, total scores between 54 and 72 as a “big problem”, and total scores between 73 and 100 as a “very big problem” [39]. The test-retest reliability of the TFI is good (r = 0.75) [38]. A decrease of 13 points or more is required to attain a clinically relevant improvement [38].

### 2.5. Potential Predictors

In this study, five potential predictors were included in our analysis based on our clinical knowledge and the previous literature: 1. gender 2. age 3. days between course initiation and completion 4. TFI score at baseline, and 5. duration of the tinnitus, dichotomized into acute tinnitus (≤3 months) and subacute and chronic tinnitus (>3 months).

### 2.6. Data Analysis

Prior to conducting the primary analyses, the normality of the data was tested with the Kolmogorov–Smirnov test. Differences between the TFI scores from baseline and follow-up (ΔTFI) were calculated to investigate the tinnitus change over time before and after completion of the Still Tinnitus course.

To address the first research question—to assess the percentage of patients that experienced a clinically meaningful improvement following the Still Tinnitus course—the clinically relevant improvement was established. For this variable, patients were categorized into two groups: those with a reduction of 13 points or more on the TFI questionnaire (i.e., clinically relevant improvement) and those with a reduction of less than 13 points on the TFI questionnaire (i.e., no clinically relevant improvement) [38]. After that, the proportion of patients demonstrating clinically relevant improvement after finishing the course was calculated.

The second objective of the study was to identify prognostic indicators that predict a clinically relevant improvement after completion of the Still Tinnitus course. We hypothesized that patients with a “younger age” and “a shorter duration of tinnitus” would be more likely to benefit from the course than older patients with a longer duration of tinnitus, making these factors potential predictors of positive outcomes. To evaluate this hypothesis, we conducted multivariate logistic regression analyses. Here, a “clinically relevant improvement” served as the dependent variable, with “age” and the “duration of tinnitus” as independent variables. In the regression analysis, the “duration of tinnitus”, dichotomized into acute tinnitus (≤3 months) and subacute and chronic tinnitus (>3 months), “sex” (females and males), “TFI baseline score”, and “days between course initiation and completion” were included as independent variables (predictors). The odds ratio’s, 95% confidence intervals and *p*-values for each independent variable were calculated.

Analyses were conducted using IBM SPSS Statistics 29. *p* values of <0.05 were considered to be statistically significant.

## 3. Results

### 3.1. Selection Process

**Figure 1:**
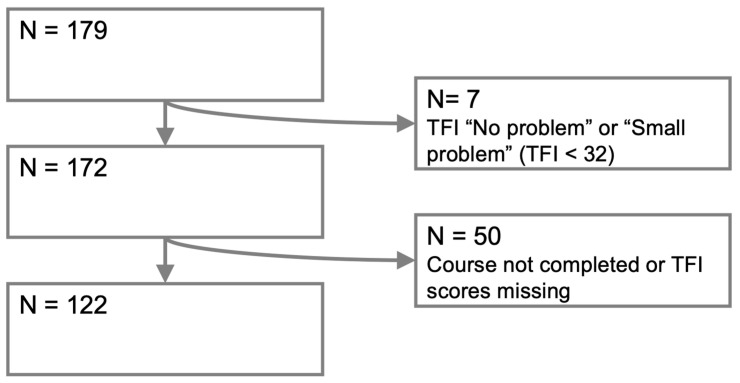
Flowchart of the patient selection process. N: number of patients, TFI: Tinnitus Functional Index.

Figure 1 provides a flowchart of the patient selection process. The documentation provided by Still Tinnitus included all of the data of the 179 patients. Of these patients, seven patients had “no problem” or a “small problem” based on the baseline TFI score. Additionally, 50 patients were excluded because it was not clear whether they completed the entire course and/or their TFI scores were missing at baseline or after the course completion. After exclusion, 122 patients met the eligibility criteria. In addition to completing the Still Tinnitus course, 14 patients had a single call with the developer of the course (RvG), 1 patient had 2 calls with the developer and 3 patients had a single call with a psychologist.

### 3.2. Patient Characteristics

The study included 122 patients who met the eligibility criteria. Sex was distributed as 59.8% male and 40.2% female. Age varied between 19 and 78, with a mean of 54.4 years (standard deviation (SD) 12.8). The mean baseline TFI score was 59.2 (SD: 14.0). Of the 122 patients, 44 experienced acute tinnitus (36.1%), while 78 experienced subacute/chronic tinnitus (63.9%). The average time between baseline and follow-up was 193.1 days (SD: 109.4). An overview of the patient characteristics is depicted in Table 1.

### 3.3. Clinically Relevant Improvement

**Figure 2:**
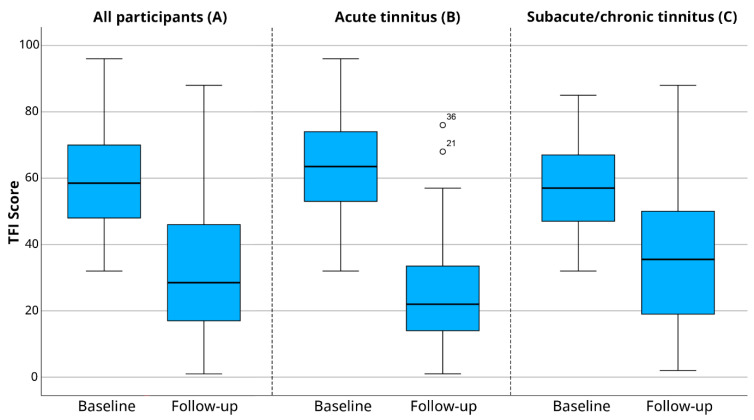
(**A**) The TFI scores at baseline and after completing the course of all patients (N = 122). TFI: Tinnitus Functional Index (**B**) The TFI scores at baseline and after completing the course of the acute tinnitus group (N = 44) (points 21 and 36 are outliers). (**C**) The TFI scores at baseline and after completing the course of the subacute/chronic tinnitus group (N = 78).

The overall reduction of the TFI score from baseline to course completion was on average 27.2 points (SD 20.8). A clinically relevant decrease, measured by a reduction of 13 points or more on the TFI questionnaire, was experienced by 91 (74.6%) patients (Table 2). Of the patients with acute tinnitus, 90.9% had a clinically relevant reduction while 65.4% of the patients with subacute/chronic tinnitus experienced a clinically relevant reduction after completing the course. Figure 2A visualizes the change in TFI score from baseline to course completion for all of the patients. Furthermore, Figure 2B shows the results at baseline and after completing the course of the acute tinnitus group, and Figure 2C of the subacute/chronic group.

### 3.4. Predictors for a Positive Outcome

The results of the multivariate logistic regression analyses are presented in Table 3. It shows that the “duration of tinnitus” (OR 4.996; 95% CI: 1.537–16.240; *p* = 0.007) and “female sex” (OR 1.912; 95% CI 0.111–7.637; *p* = 0.030) are the statistically significant predictors for a positive outcome. So, female patients and patients with acute tinnitus are more likely to have clinically relevant improvement than male patients and patients with subacute/chronic tinnitus after the course. Other variables, i.e., “age” (OR 0.966; 95% CI: 0.928–1.006; *p* = 0.099), “days between course initiation and completion” (OR 1.002; 95% CI: 0.998–1.006 *p* = 0.420), and “TFI baseline” (OR 1.007 95% CI: 0.974–1.041 *p* = 0.667) were not significant.

## 4. Discussion

The first aim of the study was to investigate the change in tinnitus severity after participation in the Still Tinnitus course. A clinically relevant average decrease of 27.2 points measured by the TFI was found. Of the 122 patients, 91 experienced a clinically relevant reduction after completion of the course. These results are in line with the findings of other eHealth studies on tinnitus. An RCT of Beukes et al. [30] compared an internet-based CBT with standard individualized face-to-face CBT. In Beukes’ study, both interventions were equally effective for the reduction in tinnitus complaints. Similar results were found by Hesser et al. [31], who compared internet-delivered CBT with internet-delivered ACT. Both groups showed a clinically relevant reduction. A systematic review of Demoen et al. [26] concluded that there is only low-to-moderate evidence that their eHealth interventions could reduce tinnitus severity. This conclusion is attributed to the high risk of bias reported in all studies included in the review.

In the analyzation of our results, we also divided patients into acute tinnitus and subacute/chronic tinnitus groups, following the recommendations of the European Guideline for Tinnitus [11]. A recent expert consensus further defines tinnitus as chronic when its duration exceeds 3 months [42]. This classification is particularly relevant, as patients with acute tinnitus have a higher likelihood of spontaneous remission compared to those with chronic tinnitus. However, a study by Wallhäusser-Franke found that only 11% of acute tinnitus patients achieved complete remission. Furthermore, the same study reported that in 30% of cases, tinnitus severity increased over the following months due to distress [43]. These findings are in line with other studies who are looking at the transition from acute to chronic tinnitus [42,44]. Given that patients with acute tinnitus may also benefit from the Still Tinnitus course, we chose not to exclude them from the study. In this way, we also increased our external validity of the study.

Examining the results across the two subgroups, both the acute tinnitus group (mean reduction of 37.6 points) and the subacute/chronic tinnitus group (reduction of 21.3 points) demonstrated a clinically relevant reduction in tinnitus severity following course completion. The findings suggest that both groups may benefit from interventions.

The second aim of the study was to identify predictors of a positive outcome after completion of the online Still Tinnitus course. We hypothesized that younger patients would derive greater benefits from this course. However, our results did not support this hypothesis, as age was not identified as a predictor of positive outcome. This finding contrasts with the outcome of the study by Theodoroff et al. [24], who found that a younger age was a significant factor in predicting a positive treatment response. A systematic review on “learning and use of eHealth among older adults” suggests that cognitive impairment and impaired hearing could act as barriers to eHealth adoption in the older population [35]. The author of the review recommended that eHealth technologies should be user-friendly to overcome such barriers. Therefore, a possible explanation for the lack of age-related differences in our study may be that the online Still Tinnitus course was easy to use for patients across all age groups.

Additionally, we hypothesized that a shorter duration of tinnitus is a predictor of positive outcome, and our findings support this hypothesis. Multiple regression analyses revealed that patients with acute tinnitus were five times more likely to benefit from the online Still Tinnitus course compared with patients with subacute/chronic tinnitus. These results align with findings from other tinnitus studies, which also identify shorter tinnitus duration as a significant factor in predicting a positive treatment response [24,36,37].

### 4.1. Strengths and Limitations

This study has some strengths and limitations. The Still Tinnitus course is a paid online course. Therefore, enrolled patients are those who actively seek treatment and believe in eHealth solutions. Consequently, patients may be more inclined to report positive results due to higher expectations and cognitive bias [45]. Additionally, patients may also encounter financial barriers in participation.

Furthermore, a convenience sample of tinnitus patients was utilized, as this approach was deemed most appropriate for our study’s objectives and is a widely used method in clinical research [46]. However, it is important to note that the findings derived from convenience sampling are generalizable only to the specific subpopulation from which the sample was drawn [47]. In this study, patients with a shorter duration of tinnitus (≤3 months) were included which might be a potential bias, as the majority of tinnitus studies typically focus on patients with subacute or chronic tinnitus.

On the other hand, this study included all adult patients, without the exclusion of psychological conditions such as anxiety or depression disorders. This is notable, as many tinnitus studies exclude patients with such characteristics, which potentially limits the generalizability of the findings. In addition, all patients received standardized therapy through a self-help tinnitus course. Of the 122 patients, 18 also had a coaching call with the developer (RvG) or a psychologist. This approach minimizes the potential influence of the patient-therapist relationship, which is often considered to be an important factor in treatment outcomes [48].

Our dataset lacks certain relevant tinnitus characteristics, such as the specific type of tinnitus experienced (e.g., constant or intermittent, unilateral or bilateral), prior or concurrent tinnitus interventions, and detailed information about tinnitus duration. Additionally, patients were not advised about hearing aid use, sound therapy, pharmacotherapy including antidepressants or anxiolytics, or face-to-face interactions with audiologists or behavioral health providers before or during the study. Furthermore, we excluded patients from the study who did not complete the course. This increases the risk of bias.

The mean patient follow-up time was 193 days. Most internet-based intervention studies have a maximum fixed treatment time of three months and a follow-up of six months to one year [26]. In this study, follow-up durations varied among patients due to differences in the time required to complete the course. This variability suggests that patients enrolled in the Still Tinnitus course may have had extended periods to apply the provided tools and strategies, potentially contributing to more favorable outcomes.

### 4.2. Recommendations for Future Studies

The presented results are explorative, and large prospective trials are needed to confirm current findings. The present study did not include a control group. To improve scientific evidence, it is recommended to follow a group that follows a “general interest course” with comparable intensity, parallel to the course patients. A randomized controlled trial with such a control group could replicate the current findings and provide higher scientific value.

Participation in this study required the patients to complete all five modules of the course. However, it is possible that completing fewer modules may have been sufficient for some individuals to achieve positive outcomes. Future research could investigate this by assessing the TFI score after each module, to determine the minimum number of modules needed for treatment efficacy. Furthermore, investigating the difference in efficacy between face-to-face and non-guided treatment groups could provide worthwhile insights.

The Still Tinnitus course incorporated CBT, TRT, ACT, and MBI. The question arises regarding which aspect contributes most to the treatment success for various patient types. Further studies should investigate the effective components to provide a more efficient online course and more-individualized care.

### 4.3. Clinical Relevance

Current findings indicate the potential of a self-paced online tinnitus treatment course. Aside from providing adequate treatment, the course has the ability to bypass various treatment barriers since it enables patients to receive treatment wherever they are and whenever they want. As a result, accessibility and availability are increased, and costs and the pressure on existing healthcare services are reduced.

To our knowledge, the Still Tinnitus course is the first self-paced online course incorporating CBT, TRT, ACT, and MBI in one package. Even though it is a newly developed course and despite several limitations, this course has shown potential as an evidence-based intervention to reduce tinnitus disability.

## 5. Conclusions

In a convenience sample of tinnitus patients, the “Still Tinnitus” course may help to reduce tinnitus severity based on a clinically relevant decrease on the TFI score. Despite the several limitations of this study, the present findings align with those of prior research. Two significant potential predictors for a positive outcome were identified, i.e., female sex and a shorter duration of tinnitus. The results highlight the importance of eHealth interventions in meeting the ongoing clinical needs of patients.

## Figures and Tables

**Table 1:** Descriptive patient characteristics (N = 122).

Characteristic	Mean
Age in years (SD)	54.4 (12.8)
Sex	
Male (N)	73 (59.8%)
Female (N)	49 (40.2%)
TFI Baseline (SD)	59.2 (14.0)
Time between baseline and follow-up in days (SD)	193.1 (109.4)
Duration of tinnitus	
Acute tinnitus ≤ 3 months (N)	44 (36.1%)
Subacute/chronic tinnitus > 3 months (N)	78 (63.9%)

Note: TFI: Tinnitus Functional Index, SD: standard deviation N: number of patients.

**Table 2:** Clinically relevant improvement of all patients, the acute tinnitus group, and the subacute/chronic group.

Patients	TFI Baseline (SD)	TFI Post (SD)	Δ TFI (SD)	% Clinically Relevant Improvement
All patients (N = 122)	59.2 (14.0)	32.1 (20.8)	−27.2 (20.8)	74.6%
Acute tinnitus group (N = 44)	63.1 (15.4)	25.5 (17.7)	−37.6 (20.4)	90.9%
Subacute/chronic tinnitus group (N = 78)	57.1 (12.8)	35.8 (21.6)	−21.3 (18.7)	65.4%

Note: TFI: Tinnitus Functional Index, Δ TFI: change in TFI score.

**Table 3:** Predictors of positive outcome based on multivariate logistic regression analysis.

Characteristics	B	S.E.	*p*	Odds Ratio [C.I. 95%]
Gender				
Male sex	Ref.			
Female sex	1.069	0.492	0.030 *	1.912 [0.111–7.637]
Age (years)	−0.034	0.021	0.099	0.966 [0.928–1.006]
Days between course initiation and completion	0.002	0.002	0.420	1.002 [0.998–1.006]
TFI baseline	0.007	0.017	0.667	1.007 [0.974–1.041]
Duration of the tinnitus				
Subacute/chronic tinnitus	Ref.			
Acute tinnitus	1.609	0.601	0.007 *	4.996 [1.537–16.240]

Note. B: regression coefficient, S.E.: standard error, *p* = *p*-value, C.I.: confidence interval, * statistical significance (*p* < 0.05).

## Data Availability

The data that support the findings of this study are available from the corresponding author, Annemarie van der Wal, upon reasonable request.
